# Is reoperation required for patients presenting with hepatic portal venous gas after gastrointestinal surgery: a review of the literature

**DOI:** 10.1080/07853890.2024.2389293

**Published:** 2024-08-08

**Authors:** Chanchan He, Junqiang Zhang, Bo Yuan, Yan Pang

**Affiliations:** Department of General Surgery, The Second Hospital of Lanzhou University, Lanzhou, China

**Keywords:** Hepatic portal venous gas, postoperative, pneumatosis intestinalis, acute gastrointestinal injury, intestinal necrosis

## Abstract

**Background and objective:**

Hepatic portal venous gas(HPVG) represents a rare radiographic phenomenon frequently linked to intestinal necrosis, historically deemed to need immediate surgical intervention. The pivotal query arises about the imperative of urgent surgery when a patient manifests HPVG after gastrointestinal surgery. This inquiry seeks to elucidate whether emergent surgical measures remain a requisite in such cases.

**Methods:**

The investigation into 14 cases of HPVG after gastrointestinal procedures was conducted through a comprehensive review of relevant literature. This methodological approach contributes to a nuanced understanding of HPVG occurrences following gastrointestinal surgery, informing clinical considerations and potential therapeutic strategies.

**Results:**

Among the 14 patients, 12 recovered and 2 died. 6 patients underwent surgical exploration, 4 with negative findings and recovered. 8 cases received conservative treatment, resulting in improvement for 5, and 1 initially treated conservatively, revealed perforation during later surgical exploration, leading to improvement, 1 case ended in mortality.

**Conclusion:**

After gastrointestinal surgery, in Computed Tomography (CT) imaging, the coexistence of HPVG and gastrointestinal dilatation, without signs of peritoneal irritation on abdominal examination, may suggest HPVG due to acute gastrointestinal injury, intestinal gas, and displacement of gas-producing bacteria. These patients can be managed conservatively under close supervision. In cases where HPVG coexists with gastrointestinal dilatation and Pneumatosis intestinalis (PI) without signs of peritoneal irritation, conservative treatment may be continued under close supervision. However, if progressive exacerbation occurs despite close monitoring and the aforementioned treatments, timely surgical exploration is deemed necessary. When HPVG is combined with signs of peritoneal irritation, prompt laparotomy and exploration are preferred.

## 1. Introduction

Hepatic Portal Venous Gas (HPVG) denotes abnormal gas accumulation in the portal vein and its branches for various reasons. This rare imaging manifestation was historically associated with intestinal ischemia and necrosis, considering emergency laparotomy due to its high mortality. In this article, we provide a summary and analysis of previously published cases of HPVG after gastrointestinal operations. Drawing insights from relevant literature, we aim to contribute to understanding diagnosis and treatment strategies for patients experiencing HPVG after gastrointestinal surgery.

## 2. Methods

**Figure 1.**
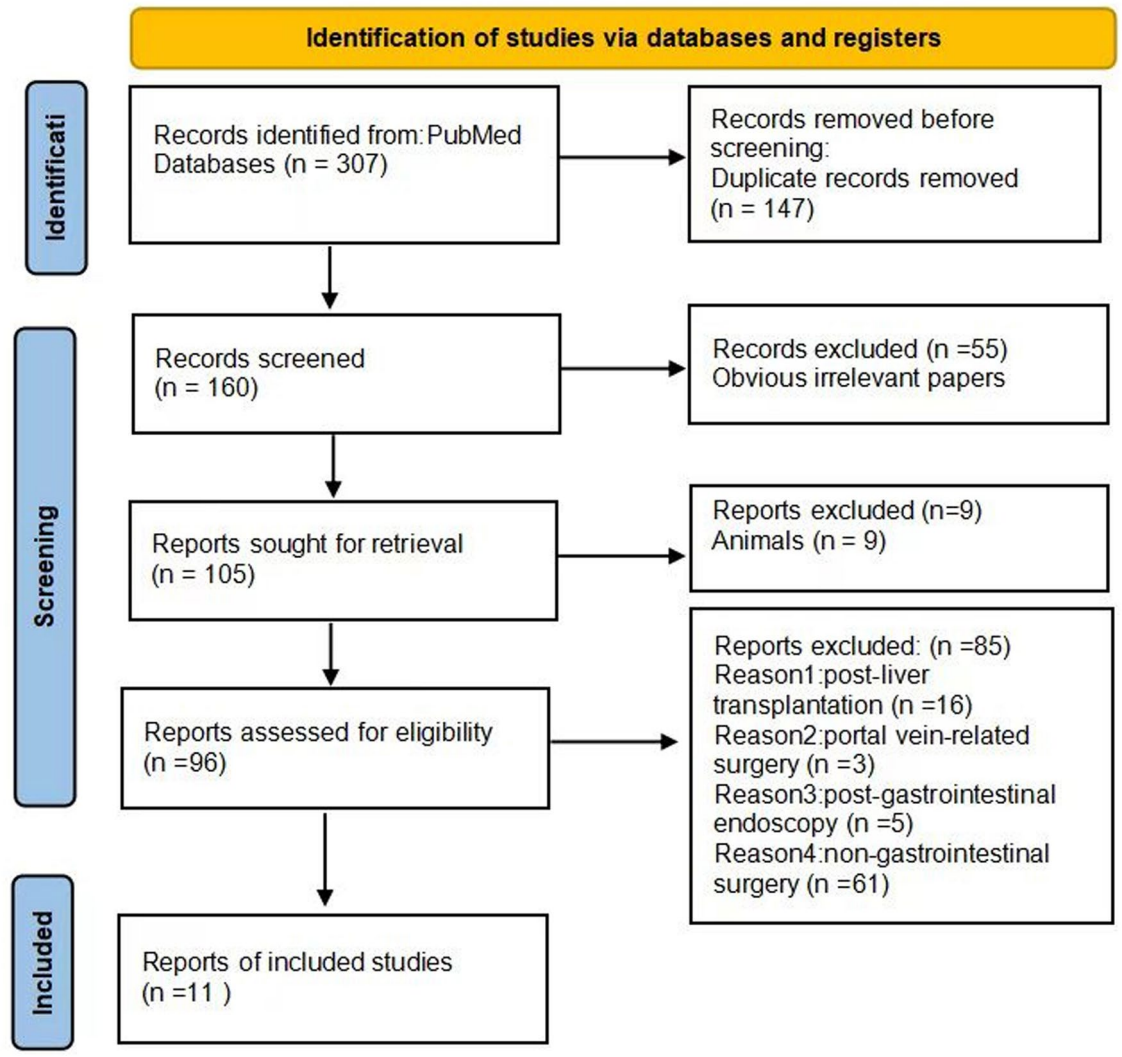
Prescription screening flow chart.

Our review adhered to the Preferred Reporting Items for Systematic Reviews and Meta-Analyses (PRISMA) standards. We conducted a literature search in PubMed using the keywords ‘(post operation) AND (portal vein gas), (gas in the portal venous blood) AND (postoperative),(portal vein gas) AND (postoperative),(portal venous gas) AND (postoperative),(post operation) AND (portal venous gas)’. The search encompassed literature from the inception of the library to 31 October 2023. All identified literature was thoroughly analyzed. Exclusion criteria comprised cases related to (1) post-liver transplantation, portal vein-related surgery, non-gastrointestinal surgery, and post-gastrointestinal endoscopy; (2) Additionally, cases lacking clear reporting of diagnostic and treatment procedures and outcomes were excluded. The literature search and screening processes are shown in Figure 1. 14 cases from 11 articles met the eligibility criteria, and details including gender, age, diagnosis, chief complaints, treatment, and evolution were recorded (Table 1) [1–11]. This information was analyzed to discern the clinical characteristics and outcomes of cases presenting with HPVG after gastrointestinal surgery.

**Table 1.** Clinical characteristics of 14 cases [1–11].

Publication year	Age(years)/sex	Diagnosis/ surgery	Post-operative days/Chief complaint	Abdominal examination	Treatment	Imaging findings	Evolution
2015 [1]	66/M	Rectal cancer/low anterior resection	2/fever, diarrheal, abdominal pain	Not mentioned	Laparotomy: negative	CT: HPVG	Recover
2019 [2]	19/M	Chronic malnutrition/open gastrostomy	10/abdominal pain, diarrhea	Not mentioned	Conservative management	CT: HPVG,PI	Recover
2017 [3]	32/M	Acute appendicitis/open appendectomy	3/fever, abdominal distension	Distended non-tender abdomen	Conservative management for 2 days, CT scan revealed a pneumoperi-toneum, ascites and peritonitis Laparotomy: revealed a jejunum perforation	CT: HPVG,PI,dilation of the small and large bowels, air within the superior mesenteric vein	Recover
2016 [4]	70/F	Bile duct carcinoma/pylorus-preserving pancreatoduodenectomy	4/abdominal distension, loss of consciousness, hypov-olemic shock	Peritoneal irritation	Laparotomy: Intestinal ischaemic necrosis	CT: HPVG,PI	Died
2016 [4]	82/M	Gastric carcinoma/ total gastrectomy (Roux-en-Y)	8/diarrhea, Multiple organ failure	Not mentioned	Conservative management	CT: HPVG,PI	Died
2016 [4]	81/M	Gastric carcinoma/ total gastrectomy (Roux-en-Y)	10/diarrhea, vomiting, abdominal distension	Not mentioned	Conservative management	CT: HPVG, dilation of the small intestine	Recover
2016 [4]	70/M	Rectal Carcinoma/abdominoperineal resection	14/steatorrhea and melena	Not mentioned	Conservative management	CT: HPVG, dilation of the gastric	Recover
2015 [5]	64/F	Sigmoid colon tumour/Hartmans procedure	6/nausea, abdominal pain.	Abdomen distend	Laparotomy: negative	CT: HPVG, PI, air within the superior mesenteric vein	Recover
2016 [6]	74/F	Colon adenocarcinoma/right hemico-lectomy	7/diarrhoea, confusion, hyponatraemic.	Negative	Laparotomy: negative	CT: HPVG, PI, air within the superior mesenteric vein	Recover
2010 [7]	58/M	Adenocarcinoma of the rectum, Small bowel torsion/sigmoidostomy	10/fever, abdominal pain, hypotension, oliguria	Pressure pain in the upper and middle abdomen	Conservative management	CT: HPVG, PI, bowel distention	Recover
2022 [8]	66/F	Gastric cancer/total gastrectomy (Roux-en-Y)	3/abdominal distention, nausea	Abdominal distension, mild tenderness, weak bowel sounds	Conservative management	CT: HPVG,PI	Recover
1975 [9]	56/F	Zollinger-Ellison syndrome/gastrectomy, esophagos-Tomy, esophageal perforation	12/abdominal pain, abdominal distention.	Abdominal distention, decreased bowel sounds	Laparotomy: nonegative	An anteroposterior supine film of the Abdomen: HPVG,PI,distended loops of small and large bowel	Recover
2005 [10]	36/F	laparoscopic Roux-en-Y gastric bypass	14/fever, vomiting, abdominal pain	not mentioned	Laparotomy: a leak was found at the gastro-jejunal anastomosis, the leak was communicating with the splenic vein	CT: HPVG	Recover
2014 [11]	22/M	Ulcerative colitis (UC) laparoscopic total colectomy with end ileostomy formation	2/abdominal pain, tachycardia, hypotension	Negative	Conservative management	CT: HPVG, PI, dilated small bowel	Recover

## 3. Results

Among the 14 cases, the age distribution ranged from 19 to 82 years, consisting of 8 males and 6 females. The primary diseases included rectal cancer, colon cancer, appendicitis, Crohn’s disease, et al. Out of the 14 cases, 12 patients experienced recovery, while 2 unfortunately died. CT imaging revealed the coexistence of HPVG with gas in the superior mesenteric vein in 3 cases, PI in 10 patients, small intestinal dilatation in 5 cases, gastric dilatation in 1 patient, and colon dilatation in 2. The main symptoms at the time of the HPVG appearance were abdominal distension, abdominal pain, vomiting, diarrhea, fever, and shortness of breath. Abdominal examinations were documented in 7 cases, indicating signs such as abdominal distension (1 case), abdominal tenderness (2 cases), and peritoneal irritation (1 case), while 2 cases showed no abdominal signs. Reoperation was conducted in 6 cases following the emergency of HPVG; 4 cases underwent negative laparotomy (all improved).8 cases were managed conservatively, with 6 showing improvement,1 case initially treated conservatively revealed pneumoperitoneum and intestinal perforation during surgery after two days of conservative treatment, leading to improvement,1 cases resulted in mortality. The overall mortality rate among the 14 cases was 14.2%.

## 4. Discussion

HPVG is a rare phenomenon characterized by specific imaging manifestations and a high mortality rate. It serves as an imaging manifestation for certain gastrointestinal diseases, abdominal infections, and other conditions, with the most common causes being intestinal ischemia and intestinal necrosis. Mechanisms leading to HPVG include [1]: (1) Increased gas in the gastrointestinal tract from various causes, resulting in high intestinal tract pressure, allowing gas to enter the bloodstream through the venous channels in the intestinal wall. Factors such as digestive tract dilatation, gastric ulceration, ulcerative colitis, Crohn’s disease, and complications of endoscopic procedures can elevate bowel lumen pressure, leading to gas escape from the mesenteric circulation into the liver vasculature; (2) Transfer of gas-producing bacteria from the intestinal tract to the bloodstream, reproducing and generating gas in the portal vein system, involving bacteria such as Escherichia coli, Klebsiella pneumoniae. Consequently, portal venous gas may manifest in benign situations under these clinical conditions. Poor recovery of gastrointestinal function postoperatively can result in severe acute gastrointestinal injury, often characterized by paralytic bowel obstruction. High intestinal lumen pressure disrupts the integrity of the gastrointestinal mucosa, enabling the invasion of gas or bacteria into the mesenteric venous plexus, eventually entering the portal vein system.

In CT imaging, HPVG is frequently observed in the left half of the liver, distinguishing it from intrahepatic bile duct pneumatosis and hepatic venous pneumatosis. HPVG may be occur together with superior mesenteric venous pneumatosis (4/14), often coinciding with gastrointestinal tract dilatation or pneumatosis intestinalis. Among these, pneumatosis intestinalis is most prevalent in the small intestine (10/14), and in severe cases, gas may extend to the stomach lining (1/14). Pneumatosis intestinalis (PI) is defined as the presence of gas within the serosal or mucosal layer of the bowel wall, typically associated with conditions like intestinal obstruction, ischemia, or necrosis.

Typical clinical symptoms in the presence of HPVG encompass abdominal distension, abdominal pain, fever, and paralytic intestinal obstruction. Some patients may exhibit respiratory abnormalities attributed to intra-abdominal hypertension and perfusion insufficiency caused by shock.

The simultaneous presence of HPVG and PI is commonly considered indicative of a high likelihood of intestinal ischemic necrosis. However, combined detection of PI with HPVG has also been associated with inflammatory, obstructive, infectious, neoplastic, traumatic, iatrogenic, and idiopathic causes [7]. In the 14 cases examined, we identified simultaneous presence of PI and HPVG in 10 patients, of whom 5 underwent additional surgery (including intestinal necrosis in 1 case, jejunum perforation in 1 case, and negative results in 3 cases), The remaining 5 cases underwent conservative treatment(4 cases were recovered and 1 died). These observations emphasize that not all instances of simultaneous HPVG and PI indicate intestinal ischemic necrosis post-gastrointestinal surgery. The determination relies on a combination of abdominal signs and clinical indicators.

While the presence of gas in the portal venous system has historically been considered a serious intra-abdominal event, advancements in imaging modalities have revealed many benign cases. The prognosis of portal venous gas once deemed poor, is now considered more closely associated with its underlying cause rather than the mere presence of gas. Although bowel ischemia is the primary mechanism for portal venous gas formation, the presence of gas itself does not provide information about the extent of bowel necrosis [1]^.^ Hussain [12] previously reported 275 cases of Hepatic Portal Venous Gas (HPVG), with etiologies encompassing intestinal ischemia and mesenteric vasculopathy (61.44%), gastrointestinal inflammation (16.26%), obstruction and dilatation (9.03%), sepsis (6.6%), medical injury and trauma (3.01%), cancer (1.8%), and primary HPVG (1.8%). Given the rising prevalence of benign cases, relying solely on CT scan results is insufficient for determining whether conservative or surgical treatment is warranted. Importantly, HPVG itself does not serve as an indication for surgery; rather, treatment and prognosis are primarily dependent on the underlying disease. For patients with HPVG, surgery is recommended at the earliest indication of intestinal ischemia, necrosis, or perforation. Alternatively, conservative treatment can be considered under close supervision [7].

Manato Fujii [13] and colleagues conducted an analysis of 30 patients with HPVG, categorized into groups with and without intestinal ischemia. Their findings indicated that the presence of signs of peritoneal irritation had a 100% predictive value for intestinal ischemia in these patients. Among the 14 cases discussed, abdominal examination details were available for 7 cases, with 1 showing signs of peritoneal irritation, subsequently confirmed as intestinal necrosis through abdominal exploration.

This review presents a comprehensive synthesis of the existing literature, delving into the treatment options for patients with HPVG after gastrointestinal surgery. But our review still has limitation, the limitation is that this review involves few cases reports which make the precise conclusions challenging. As a result, more research on the management of HPVG after gastrointestinal surgery is needed.

## 5. Conclusion

When HPVG is the sole manifestation, and vital signs are stable without signs of peritonitis, a conservative treatment approach may be adopted. This includes measures such as fasting, gastrointestinal decompression, anti-infection, and fluid rehydration under close supervision. However, given that intestinal ischemia remains a common cause of HPVG, the possibility should not be disregarded, and urgent evaluation is warranted. After gastrointestinal surgery, in CT imaging, the coexistence of HPVG and gastrointestinal dilatation, without signs of peritoneal irritation on abdominal examination, may suggest HPVG due to acute gastrointestinal injury, intestinal gas, and displacement of gas-producing bacteria. These patients can be managed conservatively under close supervision, involving gastrointestinal decompression, laxation, and broad-spectrum antibiotic use, particularly when HPVG is associated with gastrointestinal dilatation. In cases where HPVG coexists with gastrointestinal dilatation and PI without signs of peritoneal irritation, conservative treatment may be continued under close supervision. However, if progressive exacerbation occurs despite close monitoring and the aforementioned treatments, timely surgical exploration is deemed necessary. When HPVG is combined with signs of peritoneal irritation, prompt laparotomy and exploration are preferred.

## Data Availability

The data that support the findings of this study are available from the corresponding author, [He CC], upon reasonable request.
